# Engineering Decellularized Extracellular Matrix-Incorporated Apical-Out Airway Organoids

**DOI:** 10.21769/BioProtoc.5799

**Published:** 2026-08-20

**Authors:** Zhuowei Gong, Chika S. Ikpechukwu, Dhruv Bhattaram, Amy L. Ryan, Daniel J. Weiss, Xi Ren

**Affiliations:** 1 Department of Biomedical Engineering, Carnegie Mellon University, Pittsburgh, PA, USA; 2 Department of Medicine, University of Vermont, Burlington, VT, USA; 3 Department of Anatomy and Cell Biology, University of Iowa, Iowa City, IA, USA; 4 Precision Medicine Center for CF, Carver College of Medicine, University of Iowa, Iowa City, IA, USA

**Keywords:** Decellularized extracellular matrix microparticles (dECM-MPs), Apical-out airway organoids (AoAOs), Human bronchial epithelial cells (HBECs), Airway epithelial differentiation, Epithelial-ECM interactions, Whole-mount immunofluorescence

## Abstract

The airway epithelium interfaces with the external environment through its apical surface and with the extracellular matrix (ECM) through its basolateral surface. To model this organization in vitro, we developed a decellularized ECM-incorporated apical-out airway organoid (dECM-AoAO) platform in which human bronchial epithelial cells (HBECs) self-assemble around human lung-derived decellularized ECM microparticles (dECM-MPs). This configuration preserves apical-out polarity while enabling direct epithelial–ECM interactions. Here, we describe a protocol for the vacuum filtration and quantification of dECM-MPs, the generation of dECM-AoAOs, and ultimately, whole-mount immunofluorescence staining for organoid characterization.

Key features

• This protocol incorporates dECM as size-refined microparticles, enabling a consistent and reproducible ECM input.

• The workflow uses suspension culture to generate apicalout airway organoids, eliminating the need for matrix embedding while preserving ECM–cell interactions.

• dECM-MPs are directly wrapped by epithelial cells, simplifying handling and supporting native-like epithelial–ECM integration.

• The system is compatible with wholemount immunofluorescence staining for comprehensive epithelial lineage analysis.

## Background

The airway epithelium is a polarized barrier that protects the respiratory tract from inhaled pathogens and environmental particulates while sustaining mucociliary clearance. Its function relies on the coordinated activity among multiciliated, secretory, and basal stem/progenitor cells, which collectively maintain epithelial homeostasis and support injury repair [1,2]. In native tissue, the apical surface faces the airway lumen, whereas the basal surface interfaces with the extracellular matrix (ECM), a source of structural and biochemical cues that regulate epithelial survival, differentiation, polarity, signaling, and morphogenesis [3–7].

Airway organoids provide a tractable platform for modeling epithelial biology, yet current systems impose a trade-off between ECM engagement and apical accessibility. Hydrogel-embedded, apical-in organoids preserve epithelial–ECM interactions but restrict direct access to the apical surface [2,8–10]. Conversely, apical-out organoids exhibit proper surface polarization and enable direct interrogation of apical stimulation and ciliary function, but they are typically generated under ECM-free or ECM-withdrawal conditions and therefore lack the capacity to model epithelial–ECM crosstalk [11–13].

Decellularized extracellular matrix (dECM) retains tissue-specific matrix composition and provides native-like biochemical cues that support epithelial morphogenesis [14–17]. To integrate ECM exposure with apical-out polarity, we developed decellularized ECM-incorporated apical-out airway organoids (dECM-AoAOs), in which primary human bronchial epithelial cells self-assemble around size-controlled airway-derived dECM microparticles. This protocol details the preparation, vacuum filtration, and quantification of dECM microparticles, the generation of dECM-AoAOs, and downstream whole-mount staining workflows for organoid characterization.

## Materials and reagents

**Biological materials**

1. Human lung tissue, acquired from the University of Vermont (UVM)

2. Healthy donor-derived human bronchial epithelial cells (HBECs) (Lonza, catalog number: CC-2541)

3. 804G cells (a gift from Dr. Hongmei Mou, Massachusetts General Hospital and Harvard Medical School)

**Reagents**

1. Dulbecco’s phosphate-buffered saline (DPBS), 1×, without calcium and magnesium (Corning, catalog number: 21-031-CV)

2. Cell-culture-grade water (Corning, catalog number: 25-055-CV)

3. Bronchial epithelial cell growth medium (Lonza, catalog number: CC-3171)

4. A83-01 (Sigma-Aldrich, catalog number: SML0788)

5. Y-27632 (Cayman Chemical, catalog number: 129830-38-2)

6. DMH-1 (Tocris, catalog number: 4126)

7. CHIR99021 (REPROCELL, catalog number: 04000402)

8. RPMI 1640 (Corning, catalog number: 10-040-CV)

9. Fetal bovine serum (FBS) (Fisher Scientific, catalog number: FB12999102)

10. Penicillin-streptomycin (Thermo Fisher Scientific, catalog number: 15140122)

11. TrypLE Express (Gibco, catalog number: 12604013)

12. CryoStor^®^ CS10 cell freezing medium (STEMCELL Technologies, catalog number: 07930)

13. PneumaCult-ALI medium (STEMCELL Technologies, catalog number: 05001)

14. Heparin solution (STEMCELL Technologies, catalog number: 07980)

15. Hydrocortisone stock solution (STEMCELL Technologies, catalog number: 07925)

16. Paraformaldehyde (PFA), 4% (Santa Cruz Biotechnology, catalog number: sc-281692)

17. Triton X-100 (Sigma-Aldrich, catalog number: X100-500mL)

18. Tween-20 (Sigma-Aldrich, catalog number: P2287)

19. Bovine serum albumin (BSA) (Sigma-Aldrich, catalog number: 9048-46-8)

20. Mouse anti-E-cadherin antibody (1:200) (Cell Signaling Technology, catalog number: 14472S)

21. Alexa Fluor 647-conjugated donkey anti-mouse IgG (1:1,000) (Thermo Fisher Scientific, catalog number: A-31571)

22. DAPI (4′,6-diamidino-2-phenylindole) in H_2_O, 10 mg/mL (Biotium, catalog number: 40043)

23. Mouse anti-acetylated α-tubulin antibody (1:500) (Sigma-Aldrich, catalog number: T6793)

24. Mouse anti-MUC5AC antibody (1:100) (Thermo Fisher Scientific, catalog number: MA5-12178)

25. Rabbit anti-SCGB1A1 antibody (1:100) (Thermo Fisher Scientific, catalog number: PA5-78215)

26. Mouse anti-TP63 antibody (1:100) (Biocare Medical, catalog number: cm163a)

27. Alexa Fluor 488-conjugated donkey anti-rabbit IgG (1:1,000) (Thermo Fisher Scientific, catalog number: R37118)

28. N-hydroxysuccinimide-Cyanine5 (NHS-Cy5) (Tocris Bioscience, catalog number: 5436-10)

29. Biotin-NHS (Millipore Sigma, catalog number: H1759-5MG)

30. Streptavidin-488 (Thermo Fisher Scientific, catalog number: S11223)

31. Human IL-13 recombinant protein (PeproTech, catalog number: 200-13-1MG)

32. Sodium deoxycholate (Sigma-Aldrich, catalog number: S1827)

33. Sodium chloride (NaCl) (Sigma-Aldrich, catalog number: S9888)

34. Calcium chloride (Sigma-Aldrich, catalog number: C4901)

35. Deoxyribonuclease I (DNase I) from bovine pancreas (Sigma-Aldrich, catalog number: DN25)

36. Peracetic acid (Sigma-Aldrich, catalog number: 269336)

37. Gentamicin (50 mg/mL) (Thermo Fisher Scientific, catalog number: 15750060)

38. Amphotericin B (Sigma-Aldrich, catalog number: A9528)

39. Pepsin from porcine gastric mucosa (Sigma-Aldrich, catalog number: P6887)

40. 10× Phosphate-buffered saline solution (PBS, Thermo Fisher Scientific, catalog number: 70011044)

41. Hydrochloric acid (HCl) (Fisher Scientific, catalog number: SA49)

42. Sodium hydroxide (NaOH) solution (Fisher Scientific, catalog number: SS255)

43. DNeasy Blood and Tissue kit (Qiagen, catalog number: 69504)

44. Dimethyl sulfoxide (DMSO), anhydrous (Sigma-Aldrich, catalog number: 276855-100ML)

**Solutions**

1. HBEC expansion medium (see Recipes)

a. 10 mM A83-01 solution

b. 10 mM Y-27632 solution

c. 10 mM DMH-1 solution

2. 804G conditioned medium (see Recipes)

3. dECM-AoAO differentiation media (see Recipes)

a. PneumaCult-ALI complete medium

b. dECM-AoAO seeding medium

c. dECM-AoAO differentiation medium

4. Lung decellularization solutions (see Recipes)

a. Triton-X solution

b. SDC solution

c. NaCl solution

d. DNase solution

e. Peracetic acid (PAA) solution

f. Storage solution

5. Digestion and neutralization solutions (see Recipes)

a. Pepsin digestion solution

b. Neutralization solution

**Recipes**

**1. HBEC expansion medium**

*Note: For all small molecules (such as A83-01, Y-27632, and DMH-1), aliquot before freezer storage to avoid multiple freeze-thaw cycles. Do not freeze-thaw more than three times.*

**a. 10 mM A83-01 solution**

Dissolve 5 mg of A83-01 (MW: 421.52 Da) in 1.186 mL of sterile DMSO to constitute a 10 mM A83-01 stock solution. Store at -20 °C for up to 6 months.

**b. 10 mM Y-27632 solution**

Dissolve 10 mg of Y-27632 dihydrochloride (MW: 320.3 Da) in 3.122 mL of sterile DMSO to constitute a 10 mM Y-27632 dihydrochloride stock solution. Store at -20 °C for up to 6 months.

**c. 10 mM DMH-1 solution**

Dissolve 1 mg of DMH-1 (MW: 380.44) in 262.85 μL of sterile DMSO to constitute a 10 mM DMH-1 stock solution. Store at -20 °C for up to 6 months.

**d. HBEC expansion medium**

**Table:** 

Reagent	Final concentration	Volume
Bronchial epithelial cell growth basal medium	N/A	500 mL
BEGM bronchial epithelial cell growth medium SingleQuots	N/A	1 kit
10 mM A83-01	1 μM	50 μL
10 mM Y-27632	5 μM	250 μL
10 mM DMH-1	0.2 μM	10 μL
10 mM CHIR99021	0.5 μM	25 μL
Total		~506 mL

*Note: Store at 4 °C for up to 4 weeks.*

**2. 804G conditioned medium**

804G rat bladder epithelial cells are thawed and expanded in 175-cm^2^ flasks using RPMI 1640 complete medium, which serves as the base medium of 804G conditioned medium. Once confluent, the cultures are used to generate 804G-conditioned medium as described in Procedure D1.

**Table:** 

Reagent	Final concentration	Volume
RPMI 1640 with L-glutamine	N/A	267 mL
FBS	10%	30 mL
Penicillin-Streptomycin	1%	3 mL
Total		300 mL

*Note: RPMI 1640 complete medium can be stored at 4 °C for up to 3 weeks.*

**3. dECM-AoAO differentiation media**

**a. PneumaCult-ALI complete medium**

**Table:** 

Reagent	Final concentration	Volume
PneumaCult-ALI basal medium	N/A	450 mL
PneumaCult-ALI 10× supplement	N/A	50 mL
PneumaCult-ALI maintenance supplement (100×)	N/A	5 × 1 mL
Heparin solution (0.2%)	0.0004%	1 mL
Hydrocortisone stock solution (200×)	1%	2.5 mL
Penicillin-Streptomycin	1%	5 mL
Total		~513.5 mL

*Note: Aliquot 45 mL per tube and store at 4 °C for up to 3 weeks.*

**b. dECM-AoAO seeding medium (day 0 only)**

**Table:** 

Reagent	Final concentration	Volume
PneumaCult-ALI complete medium	N/A	44.973 mL
10 mM A83-01	1 μM	4.5 μL
10 mM Y-27632	5 μM	22.5 μL
Total		45 mL

**c. dECM-AoAO differentiation medium (day 2 onward)**

**Table:** 

Reagent	Final concentration	Volume
PneumaCult-ALI complete medium	N/A	44.9955 mL
10 mM A83-01	1 μM	4.5 μL
Total		45 mL

*Note: dECM-AoAO assembly begins by seeding HBECs into ultra-low attachment plates using PneumaCult-ALI complete medium supplemented with 1 μM A83-01 and 5 μM Y-27632 (referred to as the dECM-AoAO seeding medium). Y-27632 is included only on day 0 to enhance single-cell viability during the initial aggregation phase. At the first medium change on day 2, cultures are switched to dECM-AoAO differentiation medium containing PneumaCult-ALI complete medium with 1 μM A83-01 only, as Y-27632 does not show improved subsequent organoid differentiation. Medium is then replaced every other day using differentiation medium without Y-27632.*

**4. Lung decellularization solutions**

**a. Triton-X solution**

**Table:** 

Reagent	Final concentration	Volume
Triton-X	0.1%	12 mL
Penicillin-Streptomycin (10,000 U/mL) (100×)	5× (i.e., 500 U/mL)	600 mL
DI water	N/A	11,388 mL
Total		~12 L

**b. SDC solution**

**Table:** 

Reagent	Final concentration	Quantity
Sodium deoxycholate powder	2%	240 g
Penicillin-Streptomycin (10,000 U/mL) (100×)	1× (i.e., 100 U/mL)	120 mL
DI water	N/A	11,880 mL
Total		~12 L

**c. NaCl solution**

**Table:** 

Reagent	Final concentration	Quantity
Sodium chloride powder	1 M	701.28 g
Penicillin-Streptomycin (10,000 U/mL) (100×)	5× (i.e., 500 U/mL)	600 mL
DI water	N/A	11,400 mL
Total		~12 L

**d. DNase solution**

**Table:** 

Reagent	Final concentration	Quantity
Bovine pancreatic DNase	0.03 mg/mL	0.36 g
Calcium chloride powder	2 mM	2.664 g
Magnesium sulfate powder	1.3 mM	1.878 g
Penicillin-Streptomycin (10,000 U/mL) (100×)	5× (i.e., 500 U/mL)	600 mL
DI water	N/A	11,400 mL
Total		~12 L

**e. PAA solution**

**Table:** 

Reagent	Final concentration	Volume
Peracetic acid	0.1%	12 mL
Ethanol (200 Proof)	4%	480 mL
DI water	N/A	11,508 mL
Total		~12 L

**f. Storage solution**

**Table:** 

Reagent	Final concentration	Quantity
Gentamicin	50 μg/mL	12 mL
Amphotericin B	2.5 μg/mL	30 mg
Penicillin-Streptomycin (10,000 U/mL) (100×)	5× (i.e., 500 U/mL)	600 mL
1× PBS	1×	11.388 L
Total		~12 L

**5. Digestion and neutralization solutions**

**a. Pepsin digestion solution**

**Table:** 

Reagent	Final concentration	Quantity
Pepsin powder	1 mg/mL	50 mg
HCl	0.01 M	50 mL
Total		50 mL

**b. Neutralization solution**

**Table:** 

Reagent	Final concentration	Volume
NaOH	0.1 M	0.5 mL
DI water	N/A	49.5 mL
Total		50 mL

**Laboratory supplies**

1. Millipore Steriflip vacuum tube top filter, 40 μm (Millipore Sigma, catalog number: SCNY00040)

2. Nalgene rapid-flow sterile disposable filter units, 0.45-μm pore size (Thermo Fisher Scientific, catalog number: 124-004)

3. Stainless steel laboratory spatula

4. Wide-orifice pipette tips, 200 μL (Axygen, catalog number: T-205-WB-C-R-S)

5. 25 cm^2^ cell culture flask (Greiner Bio-One, catalog number: 690175)

6. 75 cm^2^ cell culture flask (Greiner Bio-One, catalog number: 658175)

7. 175 cm^2^ cell culture flask (Greiner Bio-One, catalog number: 660175)

8. 96-well cell-repellent, U-bottom microplate (Greiner Bio-One, catalog number: 650970)

9. Water bath (Fisher Scientific, catalog number: FSGPD10)

10. Incubator for Cell Culture (Fisher Scientific, catalog number: 11676604)

11. Mr. Frosty^TM^ freezing container (Thermo Fisher Scientific, catalog number: 5100-0001)

12. M-20 microplate swinging bucket rotor (Thermo Fisher Scientific, catalog number: 75003624)

13. μ-slide 18-well chambered coverslip (ibidi, catalog number: 81816)

14. Sorvall Legend X1 Centrifuge (Thermo Fisher Scientific, catalog number: 75004220)

15. EVOS M7000 imaging system (Thermo Fisher Scientific, catalog number: AMF7000)

16. ECLIPSE Ts2 Inverted Cell Culture Microscope (Nikon, N/A)

17. AXR confocal microscope (Nikon, N/A)

18. Roller Pump (Stockert-Shiley, catalog number: 10-00-00)

19. Spex Sample Prep 6775 Freezer Mill

20. Scalpel

21. Forceps

22. Scissors

23. V-shaped magnetic stir bar

24. Magnetic stir plate

## Procedure

**A. Human lung-derived dECM microparticle preparation**

**A1. Human lung lobe decellularization**

*Note: Adapted from a previously published protocol by Uhl et al. [18].*

**Figure 1.**
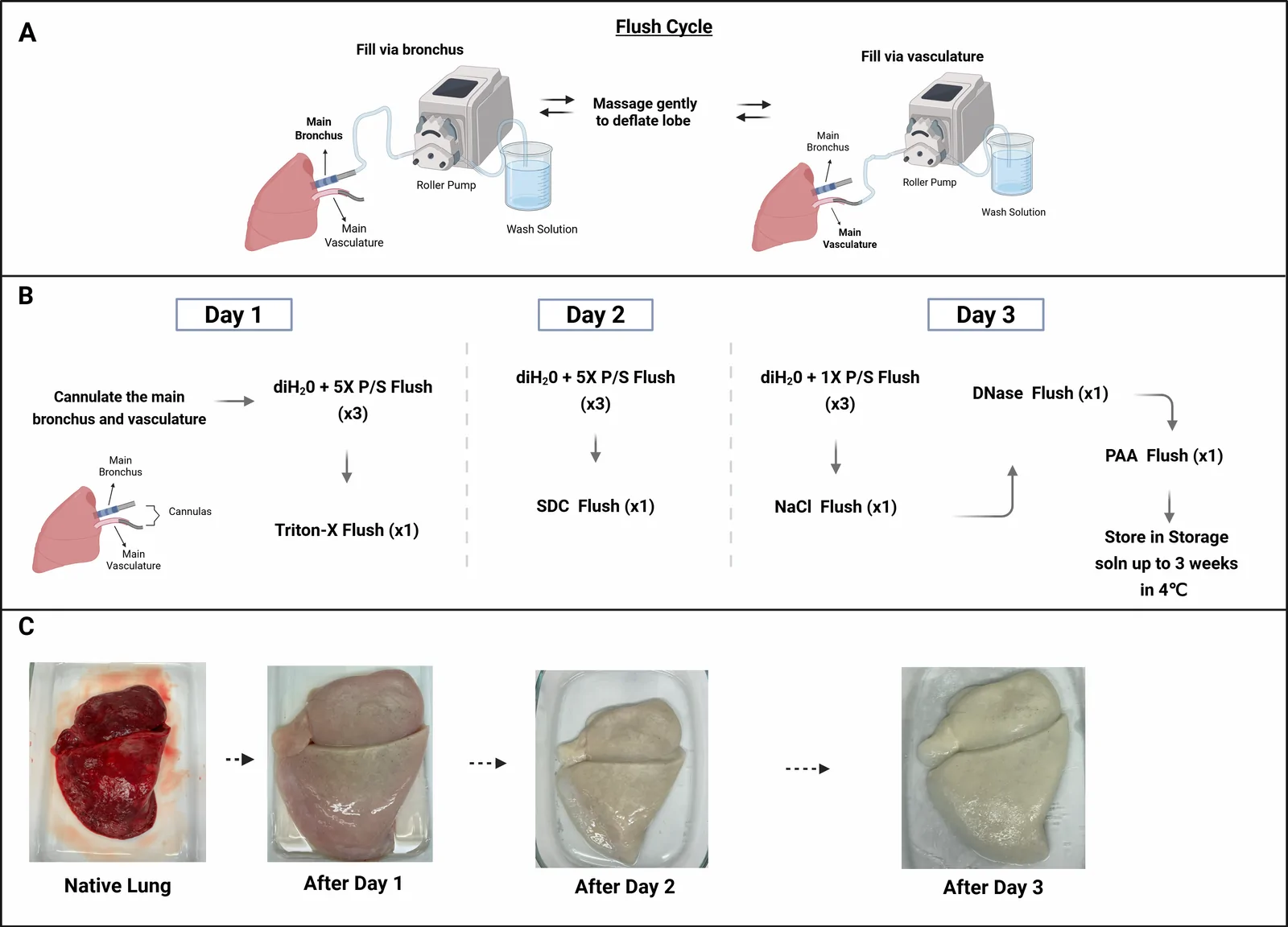
Schematic of the lung decellularization Protocol workflow. (A, B) The main bronchus and vasculature are cannulated, and a series of mild detergent solutions are perfused through both channels to remove all cellular materials over 3 days. After decellularization, the lung lobe can be stored in storage solution (soln) at 4 °C for up to 3 weeks. (C) Pictures of the lung lobe before decellularization and after each day in the decellularization process showing the gradual loss of cellular material. (Created in BioRender. Weiss Lab. (2026))

1. Perform all work under sterile conditions with sterilized utensils and solutions.

2. Dissect a single lobe by retaining as much of the main bronchi and vasculature as possible.

3. Cannulate the main bronchus and vasculature channels with appropriately sized sterilized tubing.

4. The decellularization process involves a series of mild detergent washes to effectively strip away cellular materials while preserving the architecture of the lung scaffold. Refer to Figure 1 and Table 1 for the sequence of washing and handling steps required for successful lung decellularization. For each wash solution:

a. Connect the roller pump outlet tubing to the bronchus cannula and clamp shut the vasculature.

b. Gently inflate with the wash solution by gradually increasing the flow rate to 2 L/min. Typically, 2–3 L of wash solution is sufficient to fully inflate an adult human lung lobe; however, the actual volume may vary depending on lobe size and perfusion efficiency.

c. Allow the lobe to fully deflate via passive drainage. Gentle manual manipulation (i.e., gentle kneading) can be used to help further drain the fluids.

d. Connect the roller pump outlet tubing to the vasculature cannula and clamp shut the vasculature. Repeat steps A1.4b–c.

**Table 1.** Lung decellularization volumes and conditions

	Wash solution	Purpose	Procedure	Incubation conditions
Day 1	DI water + 5× Pen/Strep		Flush through each channel thrice	
	Triton-X solution	Cell membrane disruption	Flush through each channel once. Fill both channels and submerge in solution	Overnight at 4 °C + agitation
Day 2	DI water + 5× Pen/Strep		Flush through each channel thrice	
	SDC solution	DNA and cellular proteins clearance	Flush through each channel once. Fill both channels and submerge in solution	Overnight at 4 °C + agitation
Day 3	DI water + 1× Pen/Strep		Flush through each channel thrice	
	NaCl solution	Cellular debris removal	Flush through each channel once. Fill both channels and submerge in solution	1 h at 4 °C + agitation
	DI water + 1× Pen/Strep		Flush through each channel thrice	
	DNase solution	Residual DNA digestion	Flush through each channel once. Fill both channels and submerge in solution	1 h at 4 °C + agitation
	DI water + 1× Pen/Strep		Flush through each channel thrice	
	PAA solution	Tissue sterilization	Flush through each channel once. Fill both channels and submerge in solution	1 h at 4 °C + agitation
	DI water + 1× Pen/Strep		Flush through each channel thrice	
	Storage solution		Flush both channels once. Fill both channels and submerge in solution	Up to 3 weeks at 4 °C + agitation

5. As a quality control measure, verify effective decellularization by assessing the residual DNA content using DNA quantification and gel electrophoresis.

6. Cut out some tissue chunks from various regions of the lobe and dab dry with Kimwipes.

7. Extract and quantify DNA content from the tissue using the DNeasy Blood and Tissue kit following the manufacturer's instructions.

8. Determine total DNA amount per mg of tissue. The tissue is appropriately decellularized if the DNA content is ≤50 ng/mg of tissue.

9. Run DNA samples along with a 1 kb DNA ladder and native DNA sample as a positive control on 1% agarose gel with 1× TAE buffer at 150 V until the dye front is two-thirds of the way down the gel.

10. Image the gel using a transilluminator and verify the absence of long double-stranded DNA fragments (i.e., no clear bands toward the top of the DNA ladder).

**A2. Airway dECM microparticle synthesis**

*Note: Adapted from a previously published protocol by Pouliot et al. [19].*

**Figure 2.**
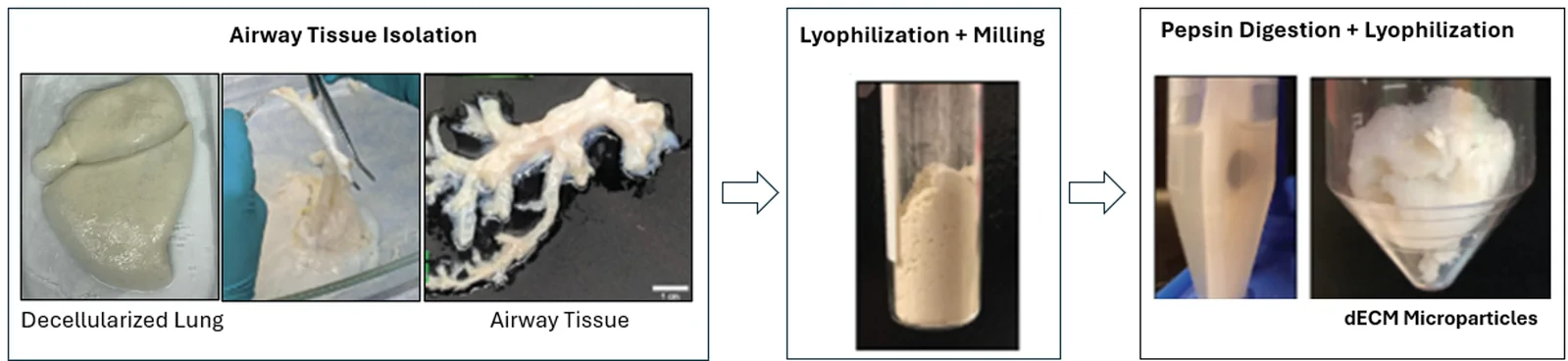
Airway dECM microparticles (dECM-MPs) synthesis workflow. Airway-enriched tissue isolated from a decellularized lung is lyophilized and milled. The milled powder is further pepsin-digested, and the soluble fraction of the digestate is collected and lyophilized to form the dECM-MPs.

1. Carefully cut out the airway tissue using sterilized surgical instruments (e.g., scalpel, scissors, and forceps), avoiding the vasculature, pleura membrane, and small airways (<1 mm) (as shown in Figure 2). The airway tree has distinctly stiffer tissue because it contains cartilaginous rings, which are absent in blood vessels.

2. Freeze the dECM tissue overnight at -80 °C.

3. Lyophilize dECM tissue for three days under constant vacuum and ≤-40 °C conditions. dECM tissue powder can be stored at -20 °C for long-term storage.

4. Mill the dECM tissue into a fine powder using a tissue miller. If using the Spex Sample Prep 6775 Freezer Mill, the following protocol can be used: Pre-cool = 1 min; Run Time = 2 min; Cool Time = 1 min; Cycle = 2; Rate = 11.

5. Resuspend dECM powder in pepsin digestion solution (1 mg/mL pepsin in 0.01 M HCl) at a 10 mg/mL concentration in a 50 mL conical tube. Keep the dECM digestion mixture below 25 mL to ensure effective digestion.

6. Place a sterilized V-shaped magnetic stir bar in the conical tube and spin on the stir plate for 72 h at room temperature.

7. Check the pH daily to ensure it remains below 2; adjust with 0.01 M HCl if necessary.

8. Check the stir bars daily to confirm continuous stirring and adjust speed if needed.

9. After digestion, centrifuge the digestate at 1,500× *g* for 20–30 min at 4 °C.

10. Transfer the supernatant to a new conical tube, avoiding ECM aggregates; record the volume.

*Tip: Try not to get any of the sediment or pellet as that can affect solubility in the later steps.*

11. Neutralize the supernatant with ice-cold 0.1 M NaOH by adding 1/9× volume of supernatant and mixing thoroughly by pipetting up and down. Avoid vortexing.

12. Confirm the pH is 7–7.4; adjust if needed with HCl and/or PBS.

13. Freeze the digestate at -80 °C overnight.

14. Once frozen, lyophilize as described earlier (step A2.3) for 2–3 days to obtain dECM microparticles (dECM-MPs). dECM-MPs can be stored at -20 °C for long-term storage. Tubes should be wrapped with parafilm to minimize moisture reabsorption.

**B. Vacuum filtration and particle counting of concentrated dECM-MP stock suspension**

1. Using a stainless-steel laboratory spatula, transfer an arbitrary amount of pre-prepared dECM microparticles (dECM-MPs) into a 1.5 mL Eppendorf tube.

*Note: Because downstream organoid assembly is normalized by particle number rather than particle mass, the starting amount of dECM-MPs used for this step does not need to be fixed.*

**Figure 3.**
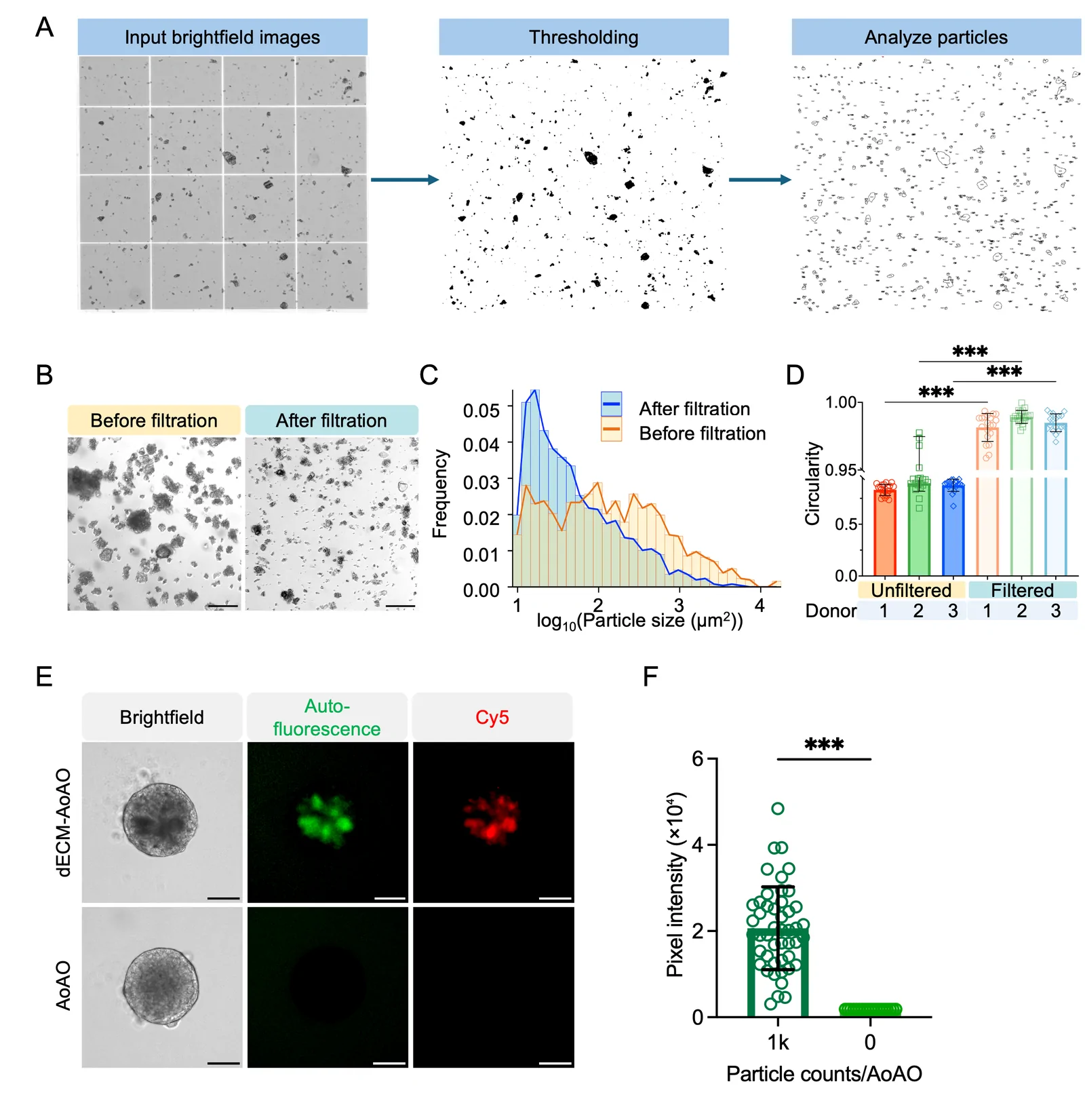
Quantification and fluorescence labeling of decellularized extracellular matrix (dECM) microparticles (MPs) for dECM apical-out airway organoid (AoAO) construction. (A) Image analysis workflow for dECM-MP quantification. Brightfield images of dECM-MPs loaded onto a hemocytometer were thresholded in Fiji and analyzed using the *Analyze Particles* function to obtain particle counts. (B) Brightfield images of dECM-MPs before and after vacuum filtration. (C) Particle size distribution on a log-transformed scale. (D) Comparison of spheroid circularity formed with filtered versus unfiltered particles across three dECM-MP donors. (E) Representative brightfield and fluorescence images of day-1 dECM-AoAOs generated with 1,000 dECM-MPs per organoid compared with ECM-free AoAOs. dECM-AoAOs assembled using NHS-Cy5-labeled dECM-MPs exhibit both intrinsic green autofluorescence and Cy5 signal. (F) Quantification of green fluorescence intensity in organoids generated with 1,000 dECM-MPs per AoAO relative to ECM-free AoAOs. Data are presented as mean ± SD. ***p < 0.001, one-way ANOVA followed by Tukey’s multiple-comparisons test (D); unpaired t-test (F). Scale bar, 100 μm. The figure is modified from Gong et al. [20] with permission under the Creative Commons Attribution License.

2. Add 1 mL of DPBS to the microcentrifuge tube to suspend the particles.

3. Transfer the particle suspension to a 50 mL conical tube containing an additional 4 mL of DPBS, for a total volume of 5 mL. Mix the suspension thoroughly by pipetting up and down approximately 30 times to obtain a homogeneous particle suspension prior to filtration.

4. Attach the 40-μm Millipore Steriflip vacuum tube top filter to the 50 mL conical tube and pass the particle suspension through the filter to remove oversized particles and refine the particle size distribution. Collect the flowthrough as the filtered dECM-MP suspension. This filtration step enriches the suspension for smaller particles and removes large ones. Filtered dECM-MPs are preferred for dECM-AoAO generation because they produce more uniform organoid morphology compared with unfiltered particles, as shown in Figure 3B–D.

5. Immediately after filtration, load 10 μL of the filtered particle suspension onto a hemocytometer.

6. Using an EVOS M7000 Imaging System, acquire 10× brightfield images from the four corner grids of the hemocytometer.

7. Open the brightfield images in Fiji for particle counting and size analysis, as illustrated in Figure 3A. For each image, crop the field to the hemocytometer counting region using *Image* > *Crop*. If the image has not been calibrated, set the pixel-to-μm conversion using *Analyze* > *Set Scale*. Segment the particles using *Image* > *Adjust* > *Threshold*, adjusting the threshold on a representative image to include visible dECM-MPs while excluding background signal. Apply the same threshold setting to all four corner-grid images from the same batch. Then use *Analyze* > *Analyze Particles* to quantify particle number and particle size. Particle size can be reported as particle area or perimeter.

*Notes:*

*1. To ensure a suitable particle size distribution for dECM-AoAO generation, confirm that the filtered dECM-MP population is mainly enriched within approximately 10–100 μm^2^ in particle area. As shown in Figure 3C, 40-μm filtration shifts the particle size distribution toward smaller particles and reduces the abundance of large particles.*

*2. Because dECM-MPs are heterogeneous in size, threshold-based counting in Fiji may include small debris or merged particles, which can bias the calculated particle concentration. To minimize this bias, use the same threshold setting for all four corner-grid images from the same batch. The threshold should be set using a representative image to segment visible dECM-MPs while excluding background signal, and the resulting segmentation mask should be visually inspected before counting. In* Analyze Particles, *apply a size-exclusion gate to remove debris and obvious merged aggregates. In this protocol, objects smaller than approximately 10 μm^2^ can be excluded as debris, whereas very large objects or visibly merged clusters should be excluded from the count. If counts vary substantially among the four grids, remix the suspension and repeat the counting step.*

8. Calculate the particle concentration of the filtered suspension using the average particle count from the four corner grids according to the following equation:

$$Particle\;concentration\;(particles/mL)=\left(\frac{Total\;particle\;count\;from\;four\;corner\;grids}{4}\right)\times 10^{4}$$

*Note: This calculation assumes the use of a standard hemocytometer, in which each large corner square has an area of 1 mm^2^ and a chamber depth of 0.1 mm, corresponding to a chamber volume of 1 × 10^-4^ mL. Therefore, the average particle count per large corner square is multiplied by 10^4^ to obtain particles/mL in the loaded suspension. In this protocol, the filtered dECM-MP suspension is loaded directly without additional dilution. If the suspension is diluted before counting, the dilution factor should be included in the concentration calculation.*

9. Store the filtered particle suspension as a concentrated dECM-MP stock at 4 °C for up to 1 month before use.

**C. Fluorescent labeling of filtered dECM-MPs (optional)**

1. If fluorescent labeling is required, add either 200 μM NHS-Cy5 or biotin-NHS to the filtered dECM-MP suspension in DPBS and incubate at room temperature for 1 h in the dark.

2. Add 5 mL of DPBS to the particle suspension and centrifuge at 220× *g* for 5 min. Carefully aspirate the supernatant to remove unbound NHS-Cy5 or biotin-NHS. Repeat this wash step twice.

3. Process the labeled dECM-MPs according to the labeling method:

a. For NHS-Cy5-labeled dECM-MPs, resuspend the washed particles directly in 5 mL of DPBS.

b. For biotin-NHS-labeled dECM-MPs, incubate the washed particles with Streptavidin-488 for 45 min at room temperature in the dark, then wash twice with DPBS to remove unbound Streptavidin-488.

*Note: dECM microparticles exhibit intrinsic green autofluorescence (Figure 3E, F), which enables rapid visualization of particle incorporation during dECM-AoAO assembly. For applications requiring more uniform or higher-contrast labeling, dECM-MPs can be fluorescently tagged using NHS-Cy5 or biotin-NHS followed by Streptavidin-488. These labeling strategies provide more consistent signal intensity than intrinsic autofluorescence and facilitate reliable confirmation of particle localization within developing organoids.*

4. After resuspending the final labeled particles in DPBS, load 10 μL of the labeled particle suspension onto a hemocytometer and repeat particle counting as described above (steps B5–8) before using the labeled dECM-MPs for organoid generation.

**D. HBEC expansion and cryopreservation**

1. Prepare 804G-conditioned medium:

a. Thaw one vial of 804G rat bladder epithelial cells in a 37 °C water bath, disinfect the vial with 70% ethanol, and transfer the cells into a biosafety cabinet.

b. Resuspend the cells in 7 mL of RPMI 1640 complete medium and centrifuge at 220× *g* for 5 min.

c. Aspirate the supernatant, resuspend the cell pellet in 1 mL of RPMI 1640 complete medium, and seed the cells into one 175-cm^2^ flask containing 25 mL of RPMI 1640 complete medium.

d. Culture the cells at 37 °C in a 5% CO_2_ incubator and replace the medium every two days.

e. Once the 804G cells reach >90% confluency, replace the medium with 50 mL of fresh RPMI 1640 complete medium.

f. Collect the conditioned medium every two days for up to four collections, replacing with 50 mL of fresh medium after each collection.

g. Pool the collected conditioned medium, filter it through a 0.45 μm filter unit, aliquot, and store at -20 °C for up to 1 year.

2. Pre-coat one 25-cm^2^ cell culture flask by adding approximately 3 mL of 804G-conditioned medium, enough to fully cover the culture surface.

3. Incubate the flask overnight at 37 °C in a 5% CO_2_ incubator.

4. On the following day, aspirate the 804G-conditioned medium.

5. Add 5 mL of DPBS to rinse the flask, then aspirate the DPBS completely.

6. Add 5 mL of HBEC expansion medium to the flask and place the flask in a 37 °C, 5% CO_2_ incubator until the thawed cells are ready to be seeded.

7. Thaw and seed one cryovial of HBECs:

a. Remove one vial of cryopreserved HBECs from storage and immediately thaw the vial in a 37 °C water bath until only a small ice crystal remains.

b. Transfer the cell suspension to a 15-mL sterile conical tube containing 7 mL of RPMI supplemented with 10% FBS and 1% penicillin-streptomycin.

c. Centrifuge the cells at 220× *g* for 5 min. Aspirate the supernatant carefully.

d. Resuspend the cell pellet in 1 mL of HBEC expansion medium and count cells.

e. Seed HBECs into the prepared 25-cm^2^ flask at approximately 3,500 cells/cm^2^, corresponding to approximately 87,500 cells per T25 flask. Return the flask to a 37 °C, 5% CO_2_ incubator.

f. Replace the medium every other day.

g. HBECs typically reach 50%–80% confluency within approximately 3–5 days after seeding.

*Note: Maintain the cells at less than 80% confluency to minimize spontaneous differentiation and senescence. Use HBECs at passages 3–5 for downstream organoid experiments. Avoid expanding the cells beyond passage 5.*

8. Passaging HBECs:

a. Aspirate the culture medium from the flask. Add 5 mL of DPBS to rinse the cells, then aspirate the DPBS completely.

b. Add 2 mL of TrypLE Express to the flask. Incubate the flask at 37 °C in a 5% CO_2_ incubator for 5 min.

c. Check cell detachment under a microscope. If most cells remain attached, continue incubating for 1 additional min. Add 7 Ml of RPMI supplemented with 10% FBS and 1% penicillin-streptomycin to neutralize TrypLE.

d. Transfer the entire cell suspension to a sterile 15-mL conical tube and centrifuge at 220× *g* for 5 min.

e. Aspirate the supernatant and resuspend the cell pellet in 1 mL of fresh HBEC expansion medium. Count cells.

f. Seed HBECs into a new 804G-conditioned 25-cm^2^ flask at approximately 3,500 cells/cm^2^ in 5 mL of prewarmed HBEC expansion medium.

9. Cryopreservation of HBECs:

a. Harvest HBECs as described in steps D8a–d.

b. After centrifugation, aspirate the supernatant completely and resuspend the cell pellet in CryoStor CS10 at approximately 500,000 cells/mL.

c. Dispense the cell suspension into cryovials at 250 μL per vial.

d. Place the cryovials into a Mr. Frosty freezing container. Store the container at -80 °C overnight.

e. On the following day, transfer the cryovials to a liquid nitrogen tank for long-term storage.

**E. Construction of dECM-AoAOs**

1. Prepare HBECs at passage 3–5 and culture them to approximately 60%–80% confluency before organoid assembly.

2. Aspirate the culture medium from the flask. For a 25-cm^2^ cell culture flask, add 1 mL of TrypLE Express and incubate the cells at 37 °C in a 5% CO_2_ incubator for 5 min to dissociate the HBECs.

3. Add 7 mL of RPMI supplemented with 10% FBS and 1% penicillin-streptomycin to neutralize TrypLE. Transfer the cell suspension to a 15-mL conical tube and centrifuge at 220× *g* for 5 min.

4. Aspirate the supernatant and resuspend the cell pellet in 1 mL of dECM-AoAO seeding medium (PneumaCult-ALI medium supplemented with 1 μM A83-01 and 5 μM Y-27632).

5. Load 10 μL of the cell suspension and count the cells using a hemocytometer to determine cell concentration.

6. For organoid assembly, use 1,000 HBECs and 1,000 dECM-MPs per organoid. In the optimized workflow, filtered dECM-MPs are loaded by particle number rather than by mass.

7. Calculate the required volumes of HBEC suspension, dECM-MP stock, and seeding medium based on the desired number of organoids using the following equations:

$$HBEC\;suspension\;volume\;(\mu L)=\frac{1000}{Cell\;concentration\;(cells/mL)}\times number\;of\;organoids\times 1000$$

$$dECM-MP\;stock\;volume\;(\mu L)=\frac{1000}{Particle\;concentration\;(particles/mL)}\times number\;of\;organoids\times 1000$$

$$dECM-AoAO\;seeding\;medium\;volume\;\left(\mu L\right)=100\;µL/well\times number\;of\;organoids-HBEC\;suspension\;volume\;\left(µL\right)-dECM-MP\;stock\;volume\;(µL)$$

8. In a sterile 15-mL conical tube, combine the calculated volumes of HBEC suspension, dECM-MP stock, and dECM-AoAO seeding medium to prepare the cell-particle mixture. Mix gently but thoroughly to ensure even distribution of cells and particles.

*Note: Maintain the final DPBS concentration at ≤1% (v/v) during organoid assembly to minimize dilution of the culture medium.*

**Figure 4.**
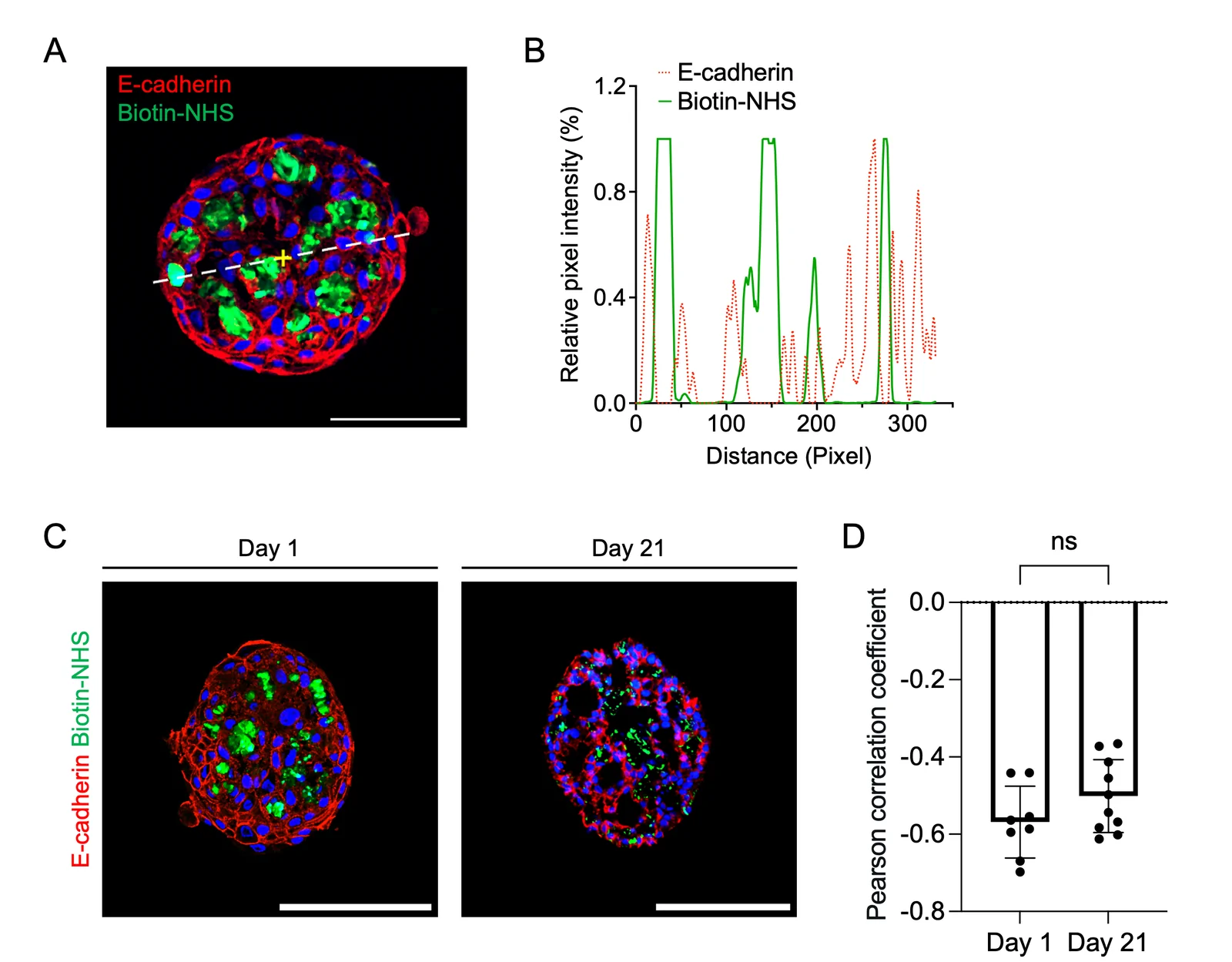
Decellularized extracellular matrix microparticle (dECM-MP) incorporation and spatial organization during dECM apical-out airway organoid (AoAO) assembly and differentiation. (A) Representative confocal image of a day-1 dECM-AoAO generated with 1,000 biotin-NHS-labeled dECM-MPs and stained for E-cadherin. The dashed line indicates the region used for line profile analysis. Scale bar, 100 μm. (B) Line profile analysis showing the relative fluorescence intensity of E-cadherin-positive epithelial cells and biotin-labeled dECM-MPs along the dashed line. (C) Representative confocal images of biotin-labeled dECM-AoAOs on day 1 and day 21, stained for E-cadherin. Scale bars, 100 μm. (D) Quantification of the Pearson correlation coefficient between E-cadherin and biotin-NHS signals. Each data point represents an individual slice. Data are presented as mean ± SD. ns, not significant; unpaired t-test. The figure is modified from Gong et al. [20] with permission under the Creative Commons Attribution License.

9. Dispense 100 μL of the HBEC/dECM-MP mixture into each well of a U-bottom, 96-well, cell-repellent plate.

10. Centrifuge the plate at 100× *g* for 5 min to bring the cells and particles to the bottom of the wells and facilitate subsequent aggregation.

11. Incubate the plate at 37 °C with 5% CO_2_ overnight to allow the cell-particle mixture to self-assemble. During assembly, dECM-MPs are typically incorporated as scattered particles within the inner region of the organoid, while HBECs progressively organize around them to form the outer epithelial structure, as shown in Figure 4.

*Note: Organoids generated with and without dECM-MPs can be compared to confirm dECM-MP incorporation, as shown in Figure 3E, F. dECM-AoAOs exhibit particle-derived green autofluorescence, whereas AoAOs generated without dECM-MPs do not.*

12. Perform half-medium changes every other day throughout differentiation. On day 2, perform the first medium change as follows:

a. Using 200-μL wide-orifice pipette tips, gently remove the entire medium containing the organoid from each well and determine the medium volume.

b. Add cell-culture-grade water to compensate for evaporation according to the following formula:

Water to add (μL) = 100 μL - measured medium volume (μL)

c. After adding water, allow the suspension to sit at room temperature for 5 min to allow thorough mixing.

d. Remove 50 μL of medium from each well and replace it with 50 μL of dECM-AoAO differentiation medium (PneumaCult-ALI medium supplemented with 1 μM A83-01 only, without Y-27632).

e. Continue differentiation for 21 days using dECM-AoAO differentiation medium, with half-medium changes every other day.

13. (Optional) Interleukin-13 (IL-13) stimulation of dECM-AoAOs

a. To stimulate dECM-AoAOs with IL-13, prepare IL-13-containing dECM-AoAO differentiation medium by adding recombinant human IL-13 to dECM-AoAO differentiation medium at 10 ng/mL.

b. On the desired treatment day, gently remove 50 μL of medium (half of the medium) from each well without disturbing the organoid. Add 50 μL of 10 ng/mL IL-13-containing differentiation medium to each well, resulting in a final IL-13 concentration in each well of 5 ng/mL in a final volume of 100 μL.

c. During the subsequent medium changes, for IL-13-treated wells, remove 50 μL of medium from each well and replace it with 50 μL of dECM-AoAO differentiation medium containing 5 ng/mL IL-13.

d. Continue IL-13 treatment with half-medium changes every other day until the desired endpoint.

*Note: In the dECM-AoAO study, IL-13 treatment was initiated on day 7 of differentiation at a final working concentration of 5 ng/mL and replenished every other day until endpoint analysis.*

**F. Whole-mount immunofluorescence staining of dECM-AoAOs**

1. Collect dECM-AoAOs from a 96-well cell-repellent plate into a 1.5-mL Eppendorf tube using 200 μL wide-orifice pipette tips.

2. Wash the organoids three times with 1 mL of DPBS containing 0.1% Tween-20 at room temperature using the following procedure:

a. Gently remove the supernatant, leaving approximately 100 μL at the bottom of the tube.

b. Add 1 mL of DPBS containing 0.1% Tween-20.

c. Let the organoids sit for 30 min at room temperature.

*Note: Inclusion of Tween-20 helps prevent the organoids from adhering to the tube wall.*

**Caution:** Avoid aspirating the organoids during washing.

3. Fix the dECM-AoAOs by gently removing the wash buffer until approximately 100 μL remains at the bottom of the tube, then add 1 mL of 4% PFA and incubate at 4 °C for 1 h.

4. After fixation, wash the organoids four times with 1 mL of DPBS containing 0.1% Tween-20, using the wash procedure described in step F2.

**Pause point:** Fixed dECM-AoAOs may be stored in DPBS containing 0.1% Tween-20 at 4 °C for later staining.

5. Permeabilize the organoids by gently removing the wash buffer until approximately 100 μL remains, then add 1 mL of DPBS containing 1% Triton X-100 and incubate for 45 min at room temperature.

6. Following permeabilization, wash the organoids four times with 1 mL of DPBS containing 0.1% Tween-20.

7. Block the organoids in 1 mL of DPBS containing 1% BSA for 20 min at room temperature.

8. Remove the blocking solution, leaving approximately 100 μL at the bottom of the tube, and add the primary antibody diluted to the desired working concentration. Incubate the samples at 4 °C overnight.

**Figure 5.**
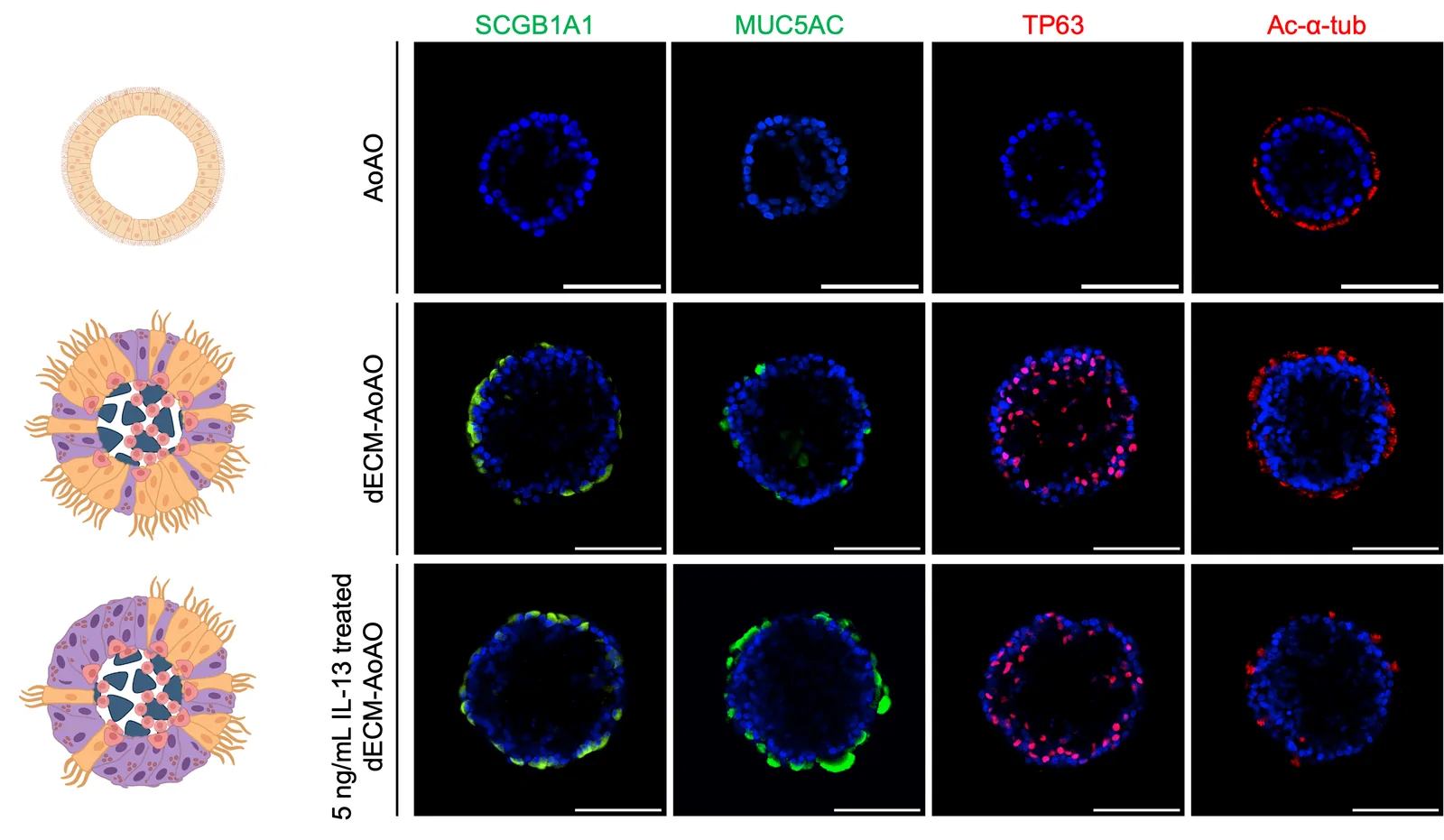
Whole-mount immunofluorescence staining of apical-out airway organoids (AoAOs) and decellularized extracellular matrix (dECM) AoAOs. Representative schematic illustrations and whole-mount confocal images showing epithelial lineage marker expression in day-21 ECM-free AoAOs, dECM-AoAOs, and dECM-AoAOs treated with 5 ng/mL IL-13. Organoids were stained for SCGB1A1^+^ club cells, MUC5AC^+^ goblet cells, TP63^+^ basal cells, and acetylated α-tubulin (Ac-α-tub)^+^ multiciliated cells. Nuclei were counterstained with DAPI. Scale bars, 100 μm. The figure is modified from Gong et al. [20] with permission under the Creative Commons Attribution License.

*Note: Primary antibodies should be selected according to the purpose of the staining. For dECM-AoAO characterization, E-cadherin can be used to label the epithelial layer (Figure 4A, C); TP63 labels basal cells; acetylated α-tubulin labels ciliated cells; SCGB1A1 labels club cells; MUC5AC labels goblet cells. These markers together allow evaluation of epithelial identity and organoid structural organization (as shown in Figure 5).*

9. Wash the organoids four times with 1 mL of DPBS containing 0.1% Tween-20.

10. Add the appropriate secondary antibody solution diluted in DPBS containing 1% BSA and incubate in the dark at room temperature for 45 min.

*Note: Although dECM-MPs may exhibit intrinsic green autofluorescence, particle-derived green autofluorescence was not readily observed after whole-mount staining of day-21 dECM-AoAOs. Therefore, 488-conjugated secondary antibodies can be used to assess epithelial cell composition in mature dECM-AoAOs. For multicolor staining, DAPI plus two antibody markers raised in two different host species can typically be assessed together in a single staining.*

11. Wash the organoids two times with 1 mL of DPBS containing 0.1% Tween-20.

12. For nuclear counterstaining, incubate the organoids in 1 mL of DPBS containing 0.1% Tween-20 supplemented with DAPI for 30 min at room temperature.

13. Wash the organoids once with 1 mL of DPBS containing 0.1% Tween-20 for 30 min, then resuspend the stained dECM-AoAOs in 1 mL of DPBS containing 0.1% Tween-20.

**Pause point:** Stained dECM-AoAOs may be stored in the dark at 4 °C for up to 2 weeks before imaging.

14. For confocal imaging, use a μ-slide 18-well chambered coverslip, which has a glass coverslip bottom. Using a wide-orifice pipette tip, transfer the stained dECM-AoAOs into the chambered well and image the organoids from the bottom using a confocal microscope.

## Validation of protocol

This protocol has been used and validated in the following research article:

• Gong et al. [20]. Recapitulating apicobasal tissue polarity in extracellular matrix-incorporated airway organoids (Figures 1–6 and Supplemental Figures S1–S13).

The HBEC culture and whole-mount immunofluorescence staining procedures are adapted from our previous apical-out airway organoid protocol, which was used and validated in the following research article:

• Wijesekara et al. [13]. Engineering rotating apical-out airway organoid for assessing respiratory cilia motility. iScience 25, 104730.

## General notes and troubleshooting

**General notes**

1. dECM-AoAO assembly is highly dependent on accurate cell and particle numbers. Always normalize organoid formation using particle counts rather than particle mass.

2. Avoid exceeding 80% confluency during HBEC expansion to prevent premature differentiation and senescence.

3. Perform half-medium changes every other day and ensure consistent medium volumes (100 μL/well) to maintain stable differentiation conditions.

**Troubleshooting**

**Problem 1:** Poor organoid formation or multi-aggregates after seeding.

Possible causes: 1) Insufficient cell-particle contact due to inadequate centrifugation. 2) Incorrect particle loading (e.g., excessive particle number relative to cells).

**Figure 6.**
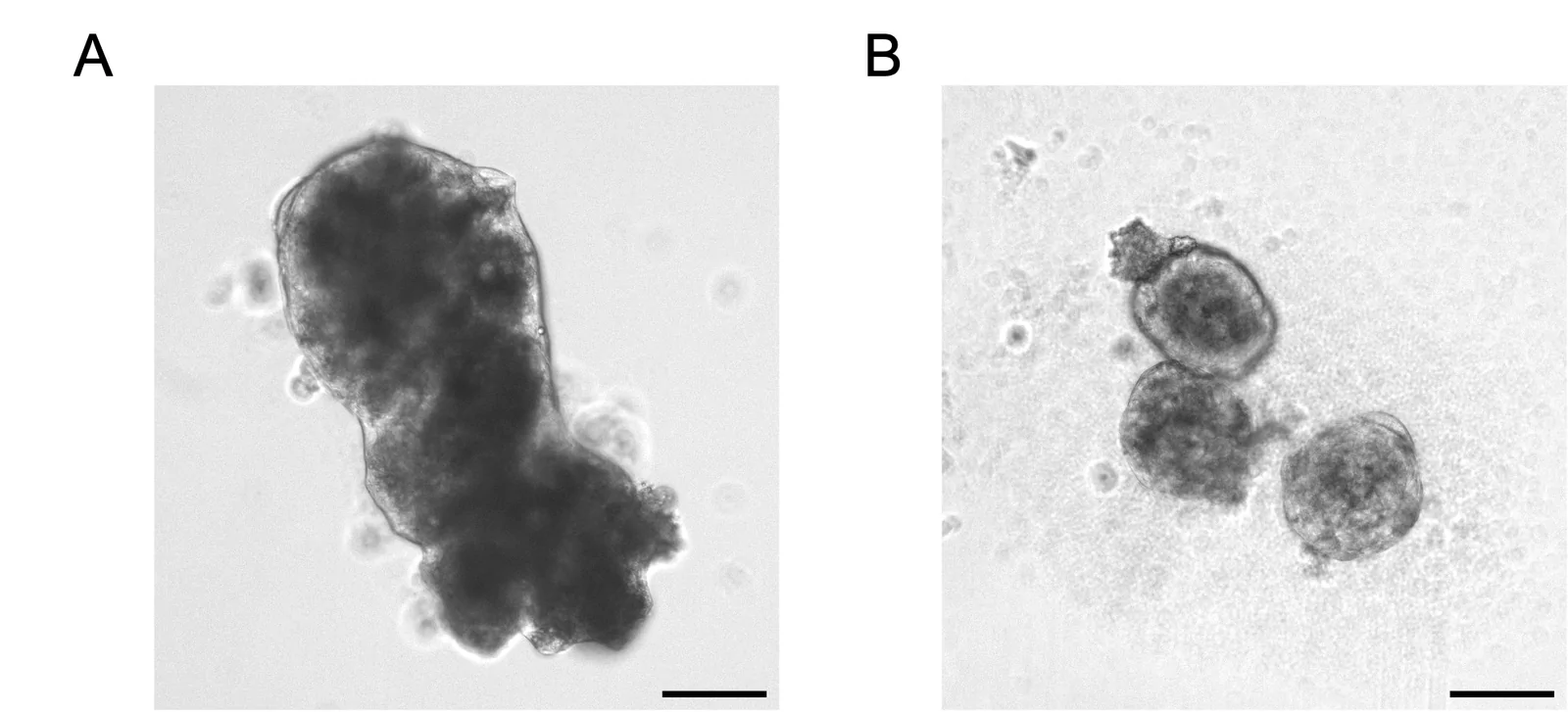
Representative brightfield images showing abnormal decellularized extracellular matrix (dECM) apical-out airway organoid (AoAO) morphology. (A) Irregularly shaped organoid with poor compaction. (B) Multi-aggregate formation, characterized by multiple separated aggregates within the same well instead of a single compact organoid. Scale bars, 100 μm.

Solutions:

1) Assess organoid morphology within the first 24–48 h after seeding. A good-quality organoid should appear as a single compact aggregate with a relatively smooth boundary (Figure 3E), whereas poor-quality cultures may show irregular aggregates or multiple separated aggregates (Figure 6).

2) Verify cell and particle concentrations prior to mixing.

3) Gently mix the cell-particle suspension thoroughly before plating.

4) Centrifuge at 100× *g* for 5 min immediately after seeding. Inspect wells under a microscope; if the cell-particle mixture is not fully collected at the bottom and in close contact, perform additional rounds of centrifugation until proper aggregation is achieved.

**Problem 2:** dECM-MP stock concentration is too low to seed 1,000 particles per organoid while maintaining the final DPBS concentration at ≤1% (v/v).

Possible causes: 1) Insufficient starting amount of dECM-MPs before filtration. 2) Particle loss during filtration, washing, or transfer. 3) Incomplete resuspension or particle settling before counting.

Solutions:

1) Before organoid assembly, calculate the volume of dECM-MP stock required to deliver 1,000 particles per organoid. If the required dECM-MP stock volume exceeds 1% of the final seeding volume, concentrate the filtered dECM-MP suspension before use.

2) To concentrate the suspension, centrifuge the filtered dECM-MPs at 500× *g* for 5 min, carefully remove part of the supernatant, and resuspend the particles in a smaller volume of DPBS. Recount the concentrated suspension before organoid assembly.

3) Mix the concentrated dECM-MP stock thoroughly by pipetting immediately before counting and before adding it to the HBEC suspension, because particles may settle quickly.

4) If the particle concentration remains too low after concentration, repeat the filtration step using a larger starting amount of dECM-MPs.

**Problem 3:** Organoid structural collapse or poor ciliary differentiation.

Possible causes: 1) Missing Y-27632 during initial seeding and incorrect medium transition. 2) HBECs at high passage numbers. 3) Low-quality or degraded A83-01 stock. 4) Over-confluent HBECs before organoid assembly.

Solutions:

1) Organoid structural collapse refers to the loss of the spherical organoid architecture, including epithelial structure fragmentation or accumulation of excessive dead cell debris. This phenotype can be identified by routine brightfield imaging during medium changes.

2) Include 5 μM Y-27632 on day 0 during seeding to improve early cell survival during spheroid formation and switch to A83-01-only medium starting on day 2 to initiate the mucociliary differentiation.

3) Use HBECs maintained in 2D culture at relatively early passage numbers. If reduced ciliary differentiation is observed, switch to an earlier passage and re-initiate dECM-AoAO culture.

4) Prepare fresh A83-01 stock if poor differentiation is observed. Store aliquoted A83-01 at -80 °C if needed and avoid repeated freeze-thaw cycles; do not freeze-thaw more than twice.
